# Supplementary material for: Cytokine-Induced Killer (CIK) Cells, In Vitro Expanded under Good Manufacturing Process (GMP) Conditions, Remain Stable over Time after Cryopreservation
Source: Pharmaceuticals (Basel). 2020 May 12;13(5):93. doi: 10.3390/ph13050093 (PMC7281026; doi:10.3390/ph13050093)
Supplement: Supplementary file 1 [file pharmaceuticals-13-00093-s001.pdf]

Supplementary Materials

Cellular growth and viability of the CIKs during expansion are illustrated in the Supplementary Materials section (Figure S1 and S2 respectively). Release criteria for drug product CIK cells are indicated in the Table S1.

QUALITY CONTROLS	ACCEPTANCE CRITERIA
Cellular Viability	>80%
Cellular Identity CD45+CD3+	>80%
CD45+CD3+CD56+	≥15%
Sterility	negative
Mycoplasma	absent
LAL test	<0,5 EU/ml

Table S1. Release criteria for drug product CIK cells

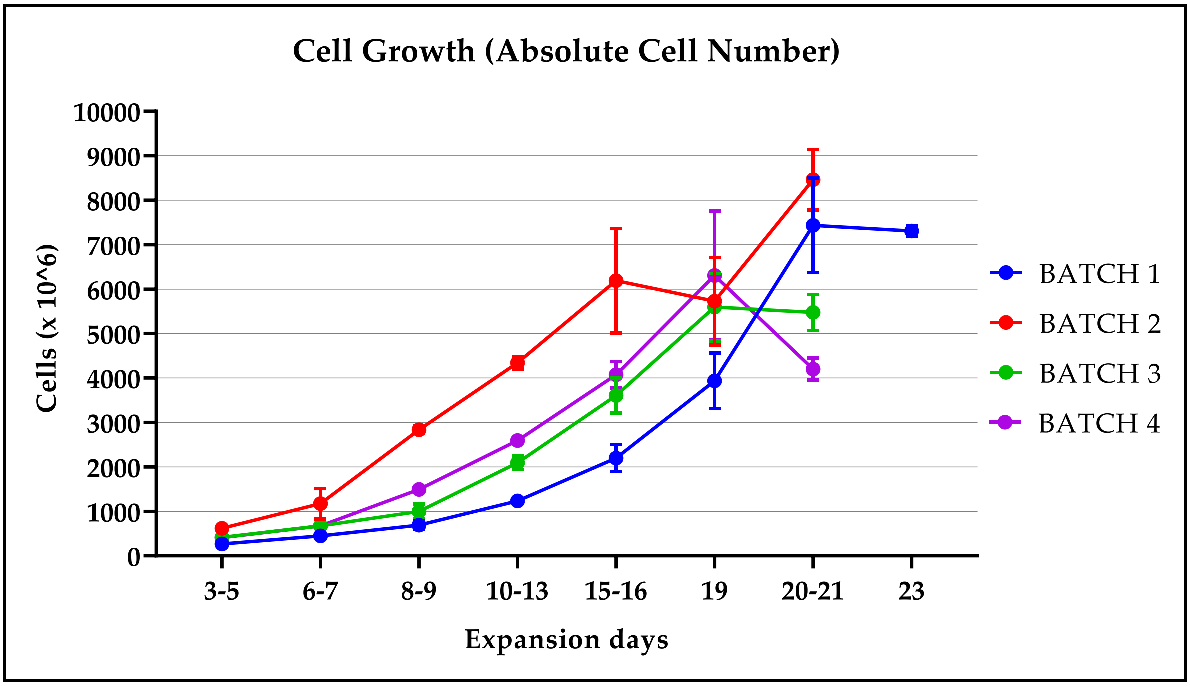

Figure S 1. Cellular growth expressed as an absolute number in millions of cells for the 4 batches during the expansion process

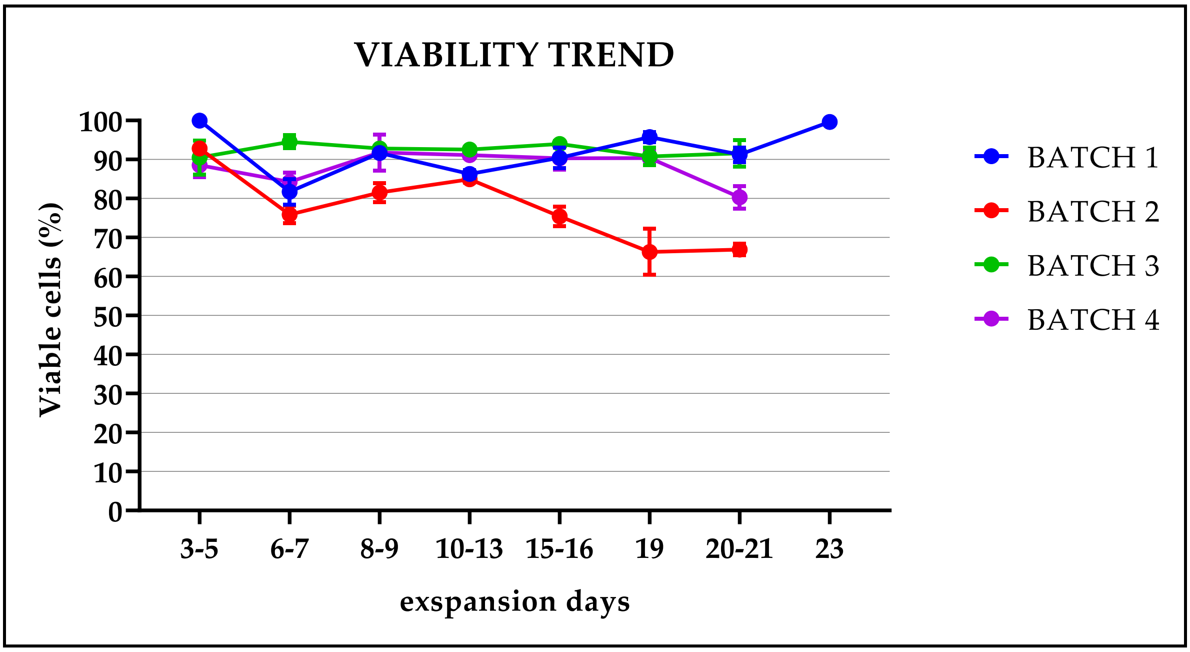

Figure S2. Viability trend expressed in percentages for the 4 batches during the expansion process
